# Supplementary material for: Detecting RNA base methylations in single cells by in situ hybridization
Source: Nat Commun. 2018 Feb 13;9:655. doi: 10.1038/s41467-017-02714-7 (PMC5811446; doi:10.1038/s41467-017-02714-7)
Supplement: Supplementary file 1 — Supplementary Information [file 41467_2017_2714_MOESM1_ESM.pdf]

Supplementary Figures

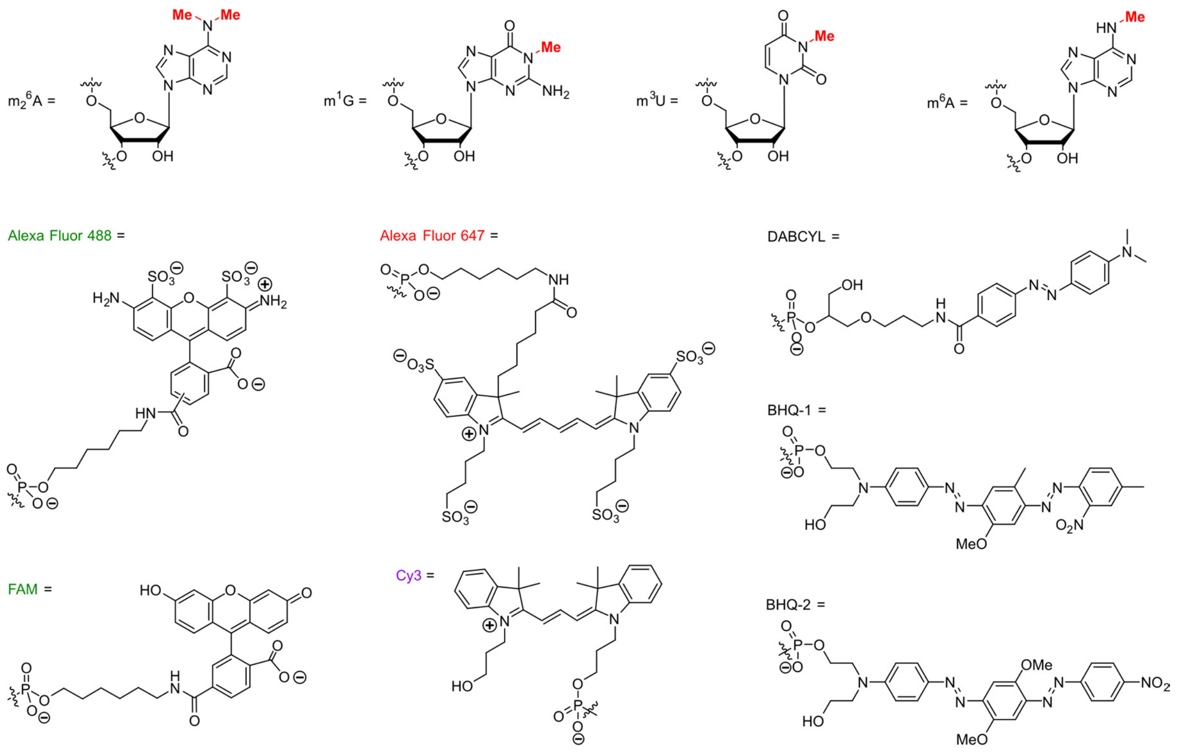

Supplementary Figure 1. Structures of methylated nucleotides used in RNA targets, as well as fluorophore and quencher labels used in Molecular Beacons. The sequences of all oligonucleotides are given in Table 1.

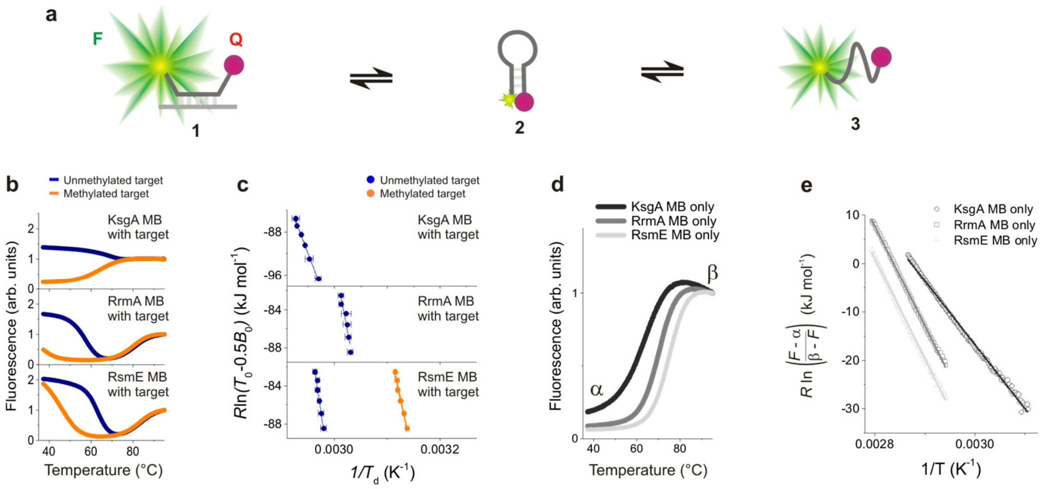

f

RNA Target	T_d	ΔT_d	$\Delta G_{1\text{-}2}$	$\Delta\Delta G_{1\text{-}2}$
KsgA MB Targets				
GUAACCGUAGGGG**AA**CCUGCGGUUGGAUCAC	66.8±0.1		50.6	
GUAACCGUAGGGG**m$_2^6$A m$_2^6$A**CCUGCGGUUGGAUCAC	n.d.	-	-	-
RrmA MB Targets				
GAACCGACUAAU**G**UUGAAAAAUUAGCG	57.1±0.6		45.1	
GAACCGACUAAU**m^{1}G**UUGAAAAAUUAGCG	n.d.	-	-	-
RsmE MB Targets				
GGGUGAAGUCG**U**AACAAGGUAACCG	62.7±0.6		56.7	
GGGUGAAGUCG**m^{3}U**AACAAGGUAACCG	45.7±0.4	17.0±0.7	34.9	21.8

g

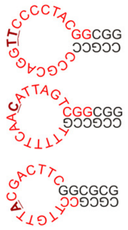

Molecular Beacon sequence		T_b	$\Delta G_{2\text{-}3}$
KsgA MB		72.4±0.1	14.7
RrmA MB		78.3±0.1	24.5
RsmE MB		82.4±0.2	26.9

Supplementary Figure 2. Thermodynamic characterization of methylation-sensitive Molecular Beacons (MBs). (a) Scheme showing phase transitions in Molecular Beacon melting in the presence of complementary RNA. **(b)** Fluorescence melting curves of methylation-sensitive beacons used for MR-FISH experiments [concentrations: 2.5 µM (KsgA MB) or 2 µM (RrmA and RsmE MBs)] in the presence of methylated and unmethylated RNA targets [concentrations: 20 µM (KsgA targets) or 25 µM (RrmA and RsmE targets)] in buffer [1× PBS (KsgA MB) or 1× PBS with 1M NaCl (RrmA and RsmE MBs)]. **(c)** Plots of $R\ln K$ vs. $1/T_d$ (the temperature at which the MB-RNA duplex is half-dissociated) at different RNA concentrations, used to calculate thermodynamic parameters of MB-RNA duplex dissociation. The mean and s.e.m. (n = 4 or 5 technical replicates) of each data point is plotted. Only RsmE MB forms a duplex with a methylated target that is sufficiently stable for measurement of T_d. **(d)** Fluorescence melting curves of methylation-sensitive beacons used in this work (2 µM) in buffer (1× PBS with 1M NaCl). **(e)** Plots of $R\ln K$ vs. $1/T$ used to calculate thermodynamic parameters of MB dissociation. **(f)** Thermodynamic parameters determined for dissociation of MB-RNA duplexes: T_d (at RNA concentrations of 20 µM RNA (KsgA MB) or 25 µM (RrmA and RsmE MBs), °C, mean ± s.e.m., n = 4 or 5 technical replicates), ΔT_d (°C, mean ± s.e.m.), $\Delta G_{1\text{-}2}$ (at 37 °C, kJ mol^{-1}) and $\Delta\Delta G_{1\text{-}2}$ (kJ mol^{-1}). Nucleotides complementary to MB sequences are shown in red. . **(g)** Thermodynamic parameters determined for dissociation of methylation-sensitive beacon hairpin loops: T_b (the temperature at which the MB hairpin loop is half-dissociated, in °C, mean ± s.e.m., n = 4 or 5 technical replicates) and $\Delta G_{2\text{-}3}$ (at 37 °C, kJ mol^{-1}). Nucleotides complementary to rRNA sequences are shown in red, with nucleotides complementary to bases methylated by the relevant methyltransferase bold and underlined.

a

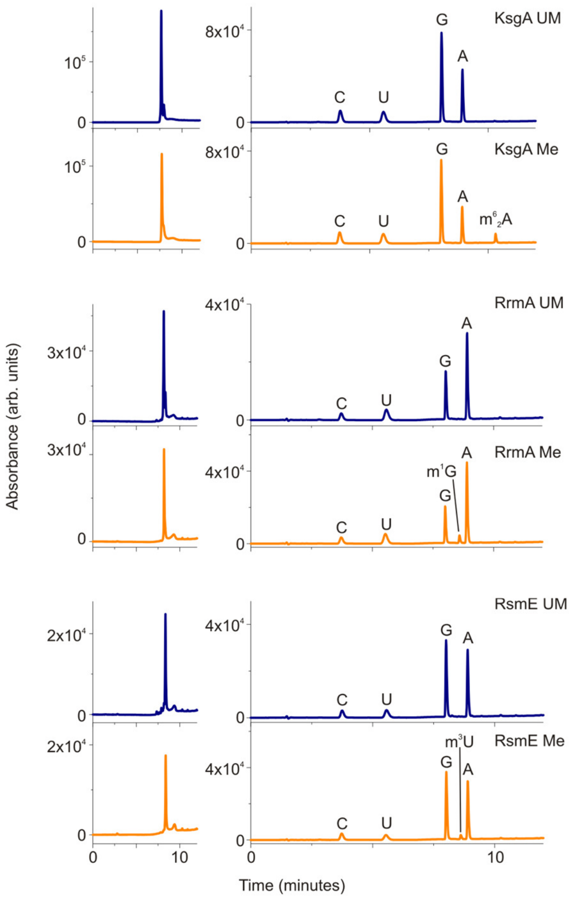

b

Nucleoside	KsgA UM		KsgA Me	
	Expected % per strand	Measured % per strand	Expected % per strand	Measured % per strand
C	22.6	22.6	22.6	22.5
U	19.4	16.7	19.4	16.2
G	35.5	40.1	35.5	39.4
A	22.6	20.6	16.1	15.2
m$^6_2$A	0.0	0.0	6.5	6.8
	**RrmA UM**		**RrmA Me**	
C	15.4	15.7	15.4	15.2
U	19.4	19.5	23.1	18.9
G	16.1	25.7	15.4	20.8
m^{1}G	0.0	0.0	3.8	6.3
A	35.5	39.0	42.3	38.7
	**RsmE UM**		**RsmE Me**	
C	16.0	16.3	16.0	16.4
U	16.0	13.8	12.0	10.1
G	36.0	39.9	36.0	39.9
m^{3}U	0.0	0.0	4.0	3.2
A	32.0	29.9	32.0	30.4

c

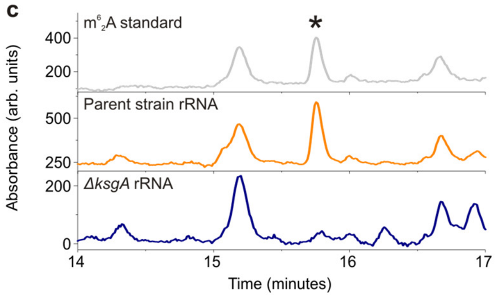

d

Nucleoside	Parent 16S rRNA		Δ*ksgA* 16S rRNA	
	Expected % per strand	Measured % per strand	Expected % per strand	Measured % per strand
C	22.6	25.3	22.6	25.8
U	20.3	20.0	20.3	20.1
G	31.3	31.8	31.3	31.4
A	25.1	22.1	25.2	22.0
m$^6_2$A	0.13	0.25	0.0	<0.1

Supplementary Figure 3. Characterization of synthetic RNA targets and rRNA by HPLC. (a) HPLC chromatograms measuring the absorbance at 254 nm of synthetic RNA oligonucleotides (UM = unmethylated, Me = methylated; sequences given in Table 1) before and after digestion and de-phosphorylation with P1 nuclease and bacterial alkaline phosphatase. Peaks are annotated with the corresponding ribonucleoside, as determined by comparison of the retention times with commercially-sourced standards. **(b)** Table showing the expected and measured composition of oligonucleotides, as determined by comparing peak areas with a dilution series prepared from commercially-sourced nucleosides. **(c)** Portion of the HPLC chromatograms measuring the absorbance at 254 nm of purified, digested and dephosphorylated samples of 16S rRNA from the parent strain (orange trace) and Δ*ksgA* (blue trace). For comparison, a commercially-sourced standard of m$^6_2$A (0.2 µM) is also shown (gray trace). The peak marked with an asterisk corresponds to m$^6_2$A with the additional peaks being unidentified background compounds. **(d)** Table showing the expected and measured compositions of 16S rRNA from the parent and *ΔksgA* strains.

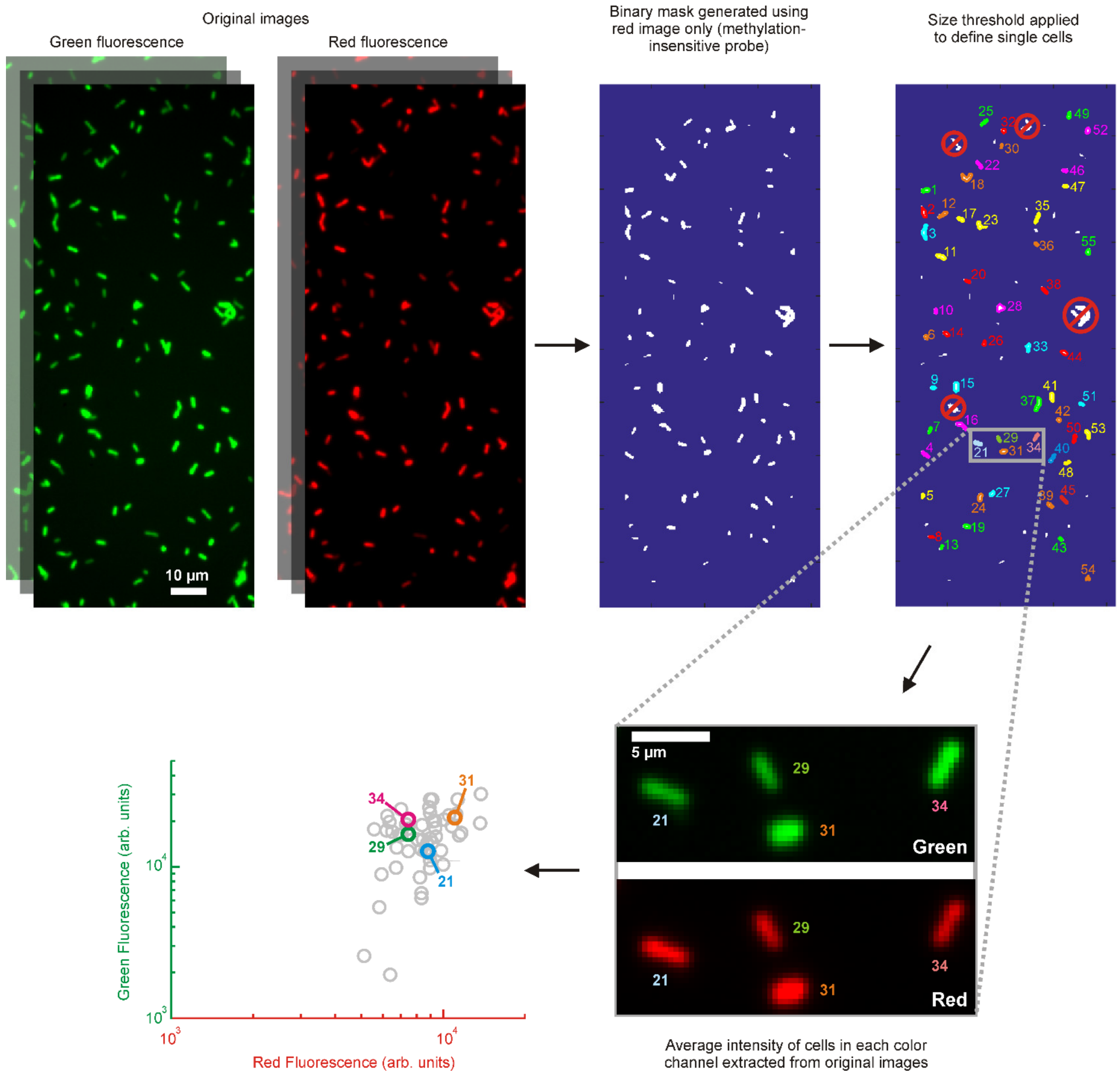

Supplementary Figure 4. Automated image analysis of MR-FISH data. The red fluorescence image resulting from hybridization of the methylation-insensitive probe) is used to generate a mask for bacteria positions by applying a bandpass filter, using an empirically-defined intensity threshold to binarize the image. From the mask, connected regions of high intensity (bacteria) are selected. These regions are then filtered according to size (areas of 1-10 µm^2 selected) and degree of homogeneity to identify discrete cells and reject clusters (examples circled in red in the top right panel). The average intensities in each color channel of the selected bacteria are then extracted.

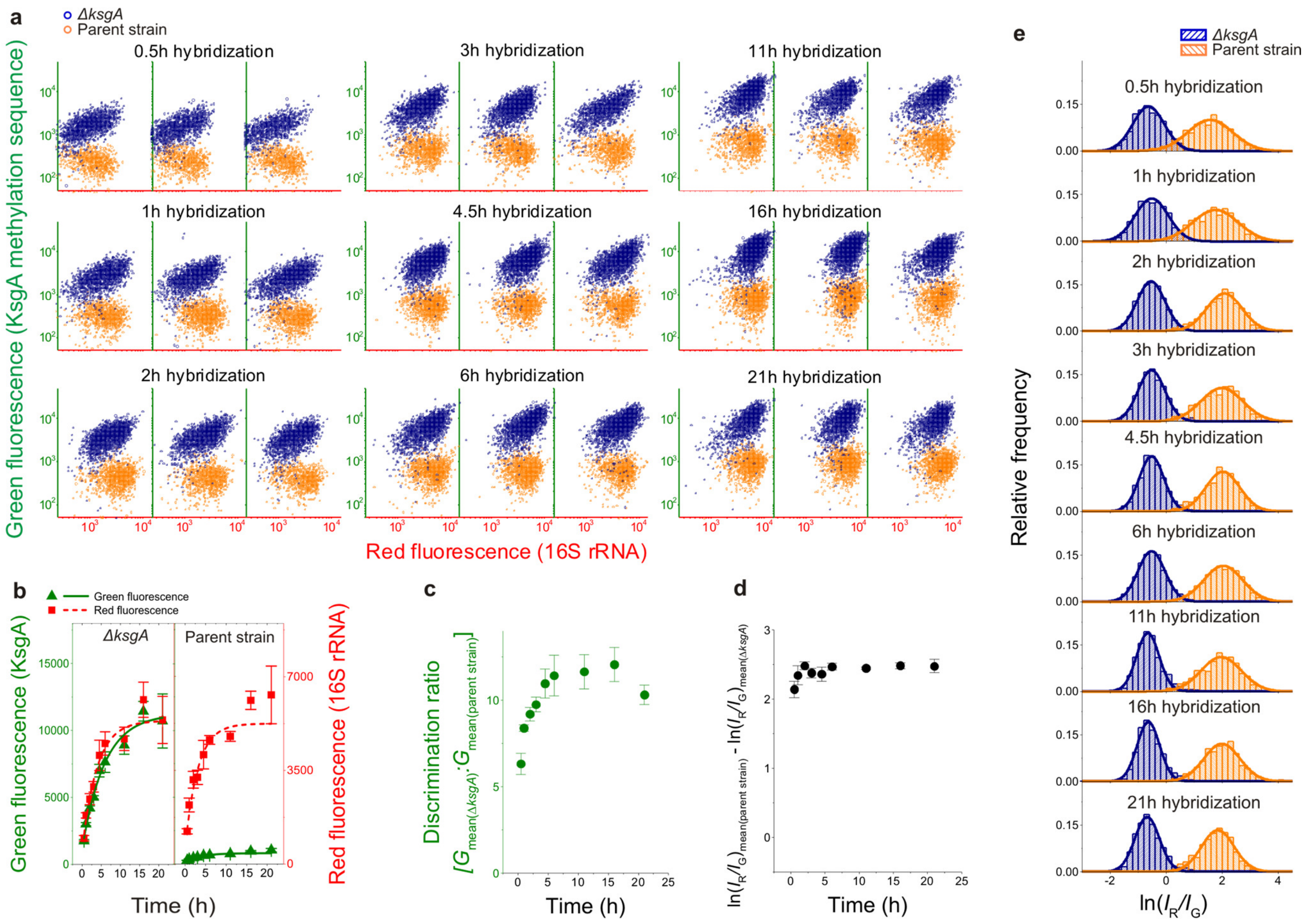

Supplementary Figure 5. Dependence of measured fluorescence parameters on duration of hybridization. (a) Scatter plots of red and green fluorescence intensities of parent strain and Δ*ksgA* bacteria stained by MR-FISH for 0.5-21 h. Data are shown for three technical replicates at each time point (n = 744-2119 cells). **(b)** Dependence of mean red and green fluorescence of parent strain and Δ*ksgA* bacteria on hybridization time. The mean and standard deviations of technical replicates (n = 3-4) are plotted. **(c)** The ratio of mean green fluorescence of Δ*ksgA* bacteria: mean green fluorescence of parent strain bacteria [$G_{\text{mean}(\Delta ksgA)}$:$G_{\text{mean(parent strain)}}$] reaches a peak of >10 after 4.5 hours of hybridization and is stable thereafter. The mean and standard deviations of technical replicates (n = 3-4) are plotted. **(d)** The difference in the log of the ratio of red:green fluorescence of parent strain and Δ*ksgA* bacteria, $\Delta\ln\left(\frac{I_R}{I_G}\right)$, i.e. $\ln\left(\frac{I_R}{I_G}\right)_{\text{mean(parent strain)}} - \ln\left(\frac{I_R}{I_G}\right)_{\text{mean}(\Delta ksgA)}$ reaches a maximum of ~2.4 after 2 hours of hybridization and is stable thereafter. The mean and standard deviations of technical replicates (n = 3-4) are plotted. (e) Histograms of the log of the ratios of red fluorescence intensity (I_R) to green fluorescence intensity (I_G) of bacteria stained by MR-FISH for 0.5-21 h.

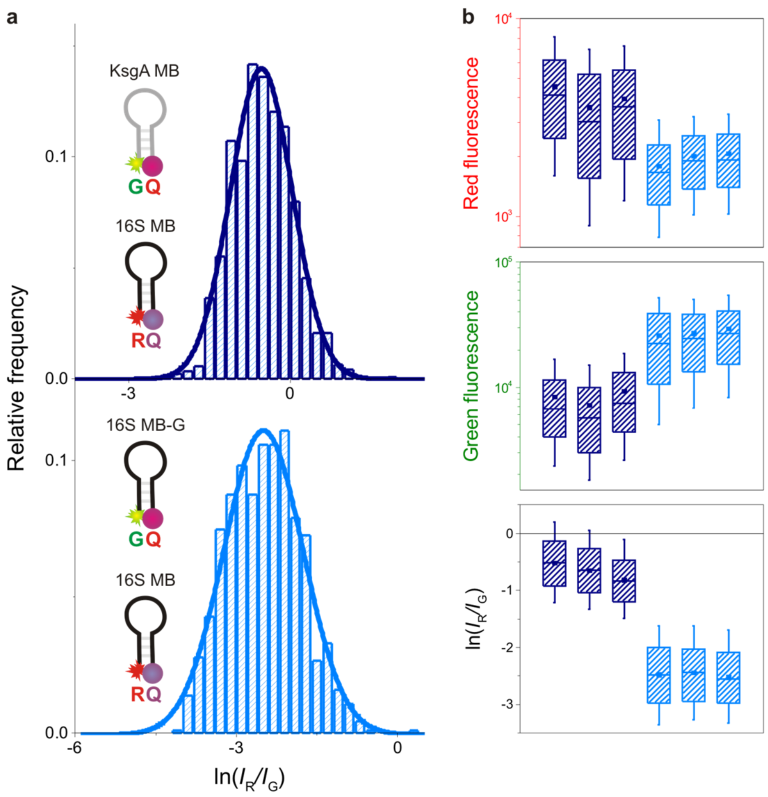

Supplementary Figure 6. The cell-to-cell variation of two-color ratios is likely dominated by signal noise. (a) Histograms of the log of the ratios of red fluorescence intensity (I_R) to green fluorescence intensity (I_G) of Δ*ksgA* bacteria stained with red and green Molecular Beacons targeting different rRNA sequences (16S MB and KsgA MB, top panel, n = 1214) or with red and green Molecular Beacons of identical sequences (16S MB and 16S MB-R, bottom panel, n = 937 cells). **(b)** Box plots of red fluorescence intensities, green fluorescence intensities and two-color ratios of technical replicates of Δ*ksgA* bacteria stained with each probe mixture (boxes: 25th-75th percentiles; whiskers: 10th-90th percentiles; horizontal lines: medians; squares: means). From left to right, n = 1214, 539, 843, 937, 1401 and 1500 cells per sample. In MR-FISH experiments with two different probes, the measured two-color ratio for any given bacterium varies according to a series of factors, which can be grouped into two categories: i) the stoichiometric ratio of the dyes within the bacterium, which depends on the hybridization efficiency of each probe and the number of binding sites, and ii) the signal detected in each channel for each bacterium, which depends on the number of fluorophores, the local illumination intensity (which is inhomogeneous across the field of view and different for each laser), the amount of autofluorescence in each channel and the quantum yield for each dye, which in turn is affected by stochastic photophysical phenomena such as quenching, blinking and photobleaching. The convolution of all these factors gives rise to a lognormal distribution of ratios. To assess the relative contributions of stoichiometry and detection factors to the widths of these distributions, we compared the ratios from MR-FISH experiments to those obtained from FISH with two probes of identical sequence, but labeled with either red or green fluorophores. These two probes compete for the same binding site, so should display minimal variation in stoichiometry from bacterium to bacterium, but will be subject to similar signal noise as MR-FISH experiments. The standard deviation of the fitted Gaussian function of two-color ratios for this sample (σ = 0.69±0.04) is slightly larger than for the MR-FISH sample (σ = 0.56±0.02), suggesting that the width of two-color ratio distributions is dominated by signal noise rather than variation in stoichiometry.

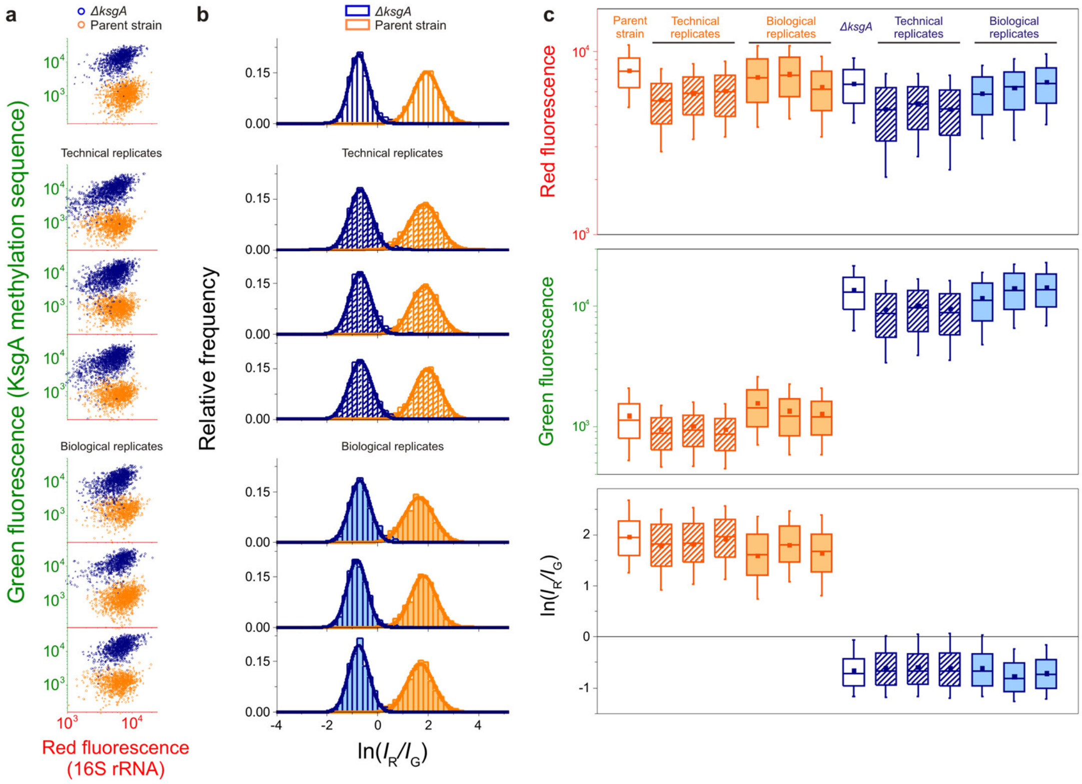

Supplementary Figure 7. MR-FISH detection of m$^6_2$A in technical and biological replicates. **(a)** Scatter plots of red and green fluorescence intensities of technical and biological replicates of parent strain and Δ*ksgA* bacteria stained for 21 h [in each panel (from top to bottom), n = 1315 (parent strain) and 1112 (Δ*ksgA*), 1056 (parent strain) and 1291 (Δ*ksgA*), 1173 (parent strain) and 1523 (Δ*ksgA*), 1115 (parent strain) and 1374 (Δ*ksgA*), 865 (parent strain) and 1118 (Δ*ksgA*), 1253 (parent strain) and 850 (Δ*ksgA*), 927 (parent strain) and 988 (Δ*ksgA*) cells per sample] **(b)** Histograms of the log of the ratios of red fluorescence intensity (I_R) to green fluorescence intensity (I_G) of technical and biological replicates of parent strain and Δ*ksgA* bacteria shown in panel **(a)**. **(c)** Box plots of red fluorescence intensities, green fluorescence intensities and two-color ratios shown in panel **(a)** (boxes: 25th-75th percentiles; whiskers: 10th-90th percentiles; horizontal lines: medians; squares: means). As expected, the distributions of red fluorescence imparted by the methylation-insensitive Molecular Beacon for all data sets overlap, while the distributions of green fluorescence imparted by the methylation-sensitive Molecular Beacon are different for parent strain and Δ*ksgA* bacteria. The two-color ratio ln(I_R)/(I_G) varies least between technical and biological replicates and offers the best discrimination between parent strain and Δ*ksgA* cells.

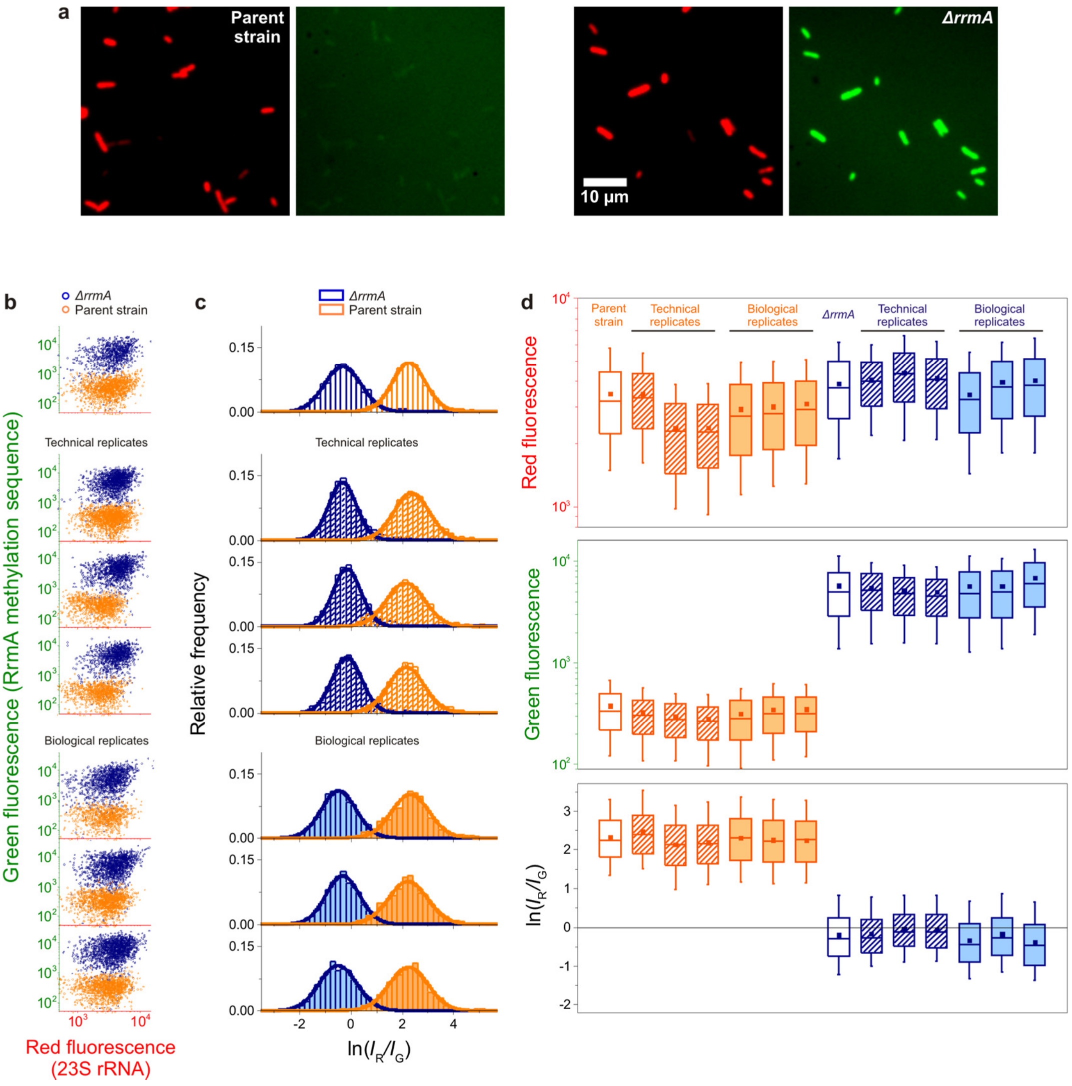

Supplementary Figure 8. MR-FISH detection of m^{1}G in technical and biological replicates. **(a)** Fluorescence images (red: left panel of each pair; green: right panel of each pair), showing that Δ*rrmA E. coli* are stained green and red by MR-FISH probes, while parent-strain cells are brightly stained only by the methylation-insensitive red probe. Scale bar: 10 µm. **(b)** Scatter plots of red and green fluorescence intensities of technical and biological replicates of parent strain and Δ*rrmA* bacteria stained for 21 h [in each panel (from top to bottom), n = 1567 (parent strain) and 966 (Δ*rrmA*), 1790 (parent strain) and 1480 (Δ*rrmA*), 1145 (parent strain) and 1275 (Δ*rrmA*), 1012 (parent strain) and 1111 (Δ*rrmA*), 843 (parent strain) and 1204 (Δ*rrmA*), 1440(parent strain) and 1484 (Δ*rrmA*), 1361 (parent strain) and 1653 (Δ*rrmA*) cells per sample]. **(c)** Histograms of the log of the ratios of red fluorescence intensity (I_R) to green fluorescence intensity (I_G) of technical and biological replicates of parent strain and Δ*rrmA* bacteria shown in panel **(b)**. **(d)** Box plots of red fluorescence intensities, green fluorescence intensities and two-color ratios shown in panel **(b)** (boxes: 25th-75th percentiles; whiskers: 10th-90th percentiles; horizontal lines: medians; squares: means). As expected, the distributions of red fluorescence imparted by the methylation-insensitive Molecular Beacon overlap, while the distributions of green fluorescence imparted by the methylation-sensitive Molecular Beacon are different for parent strain and Δ*rrmA* bacteria.

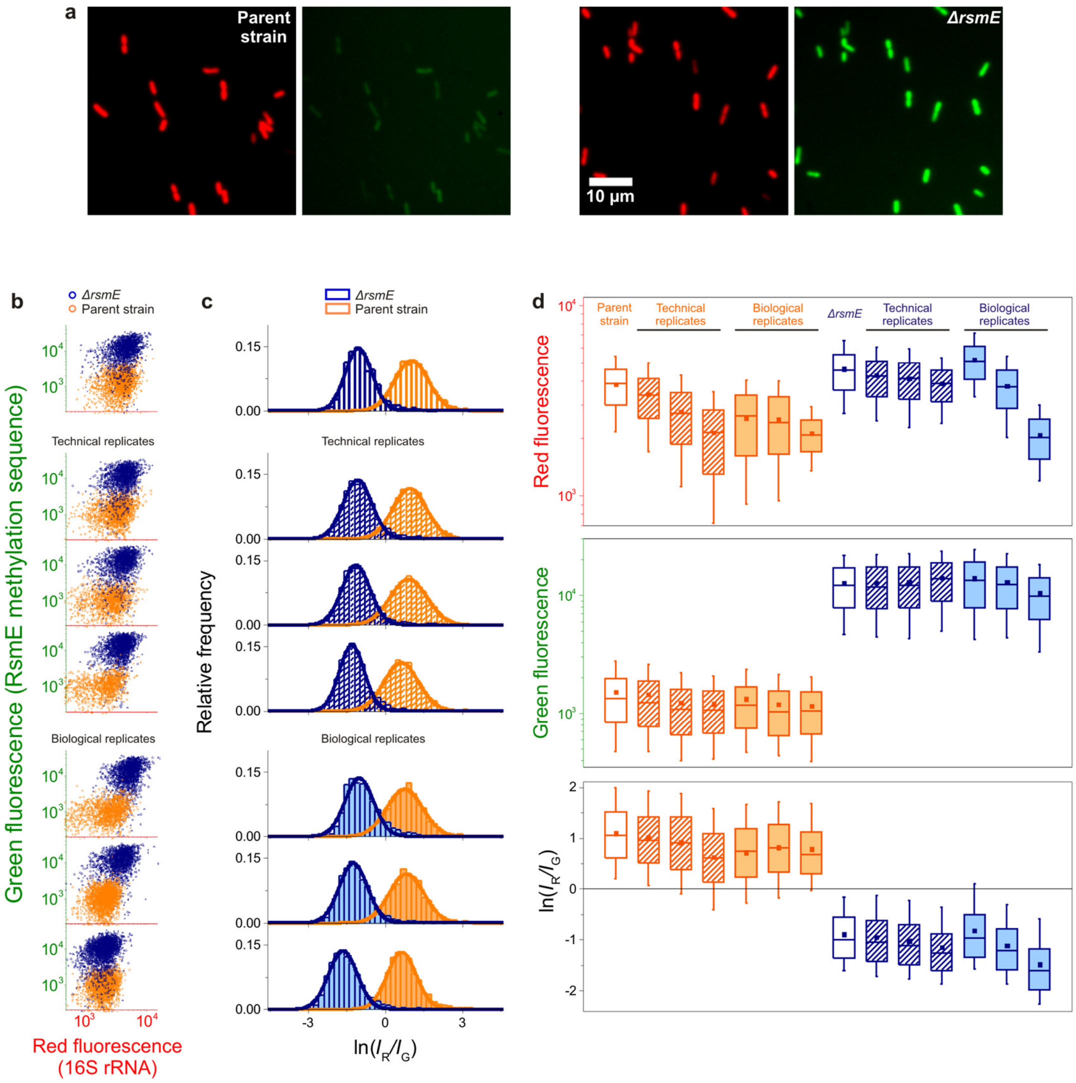

Supplementary Figure 9. MR-FISH detection of m^3U in technical and biological replicates. (a) Fluorescence images (red: left panel of each pair; green: right panel of each pair) showing that Δ*rsmE E. coli* are stained green and red by MR-FISH probes, while parent-strain cells are brightly stained only by the methylation-insensitive red probe. Scale bar: 10 μm. **(b)** Scatter plots of red and green fluorescence intensities of technical and biological replicates of parent strain and Δ*rsmE* bacteria stained for 18 h [in each panel (from top to bottom), *n* = 2266 (parent strain) and 1894 (Δ*rsmE*), 1935 (parent strain) and 1600 (Δ*rsmE*), 1445 (parent strain) and 1542 (Δ*rsmE*), 1147 (parent strain) and 1900 (Δ*rsmE*), 1155 (parent strain) and 1741 (Δ*rsmE*), 1452 (parent strain) and 1758 (Δ*rsmE*), 2450 (parent strain) and 2196 (Δ*rsmE*) cells per sample]. **(c)** Histograms of the log of the ratios of red fluorescence intensity (I_R) to green fluorescence intensity (I_G) of technical and biological replicates of parent strain and Δ*rsmE* bacteria shown in panel **(b)**. **(d)** Box plots of red fluorescence intensities, green fluorescence intensities and two-color ratios shown in panel **(b)** (boxes: 25th-75th percentiles; whiskers: 10th-90th percentiles; horizontal lines: medians; squares: means). As expected, the distributions of red fluorescence imparted by the methylation-insensitive Molecular Beacon overlap, while the distributions of green fluorescence imparted by the methylation-sensitive Molecular Beacon are different for parent strain and Δ*rsmE* bacteria.

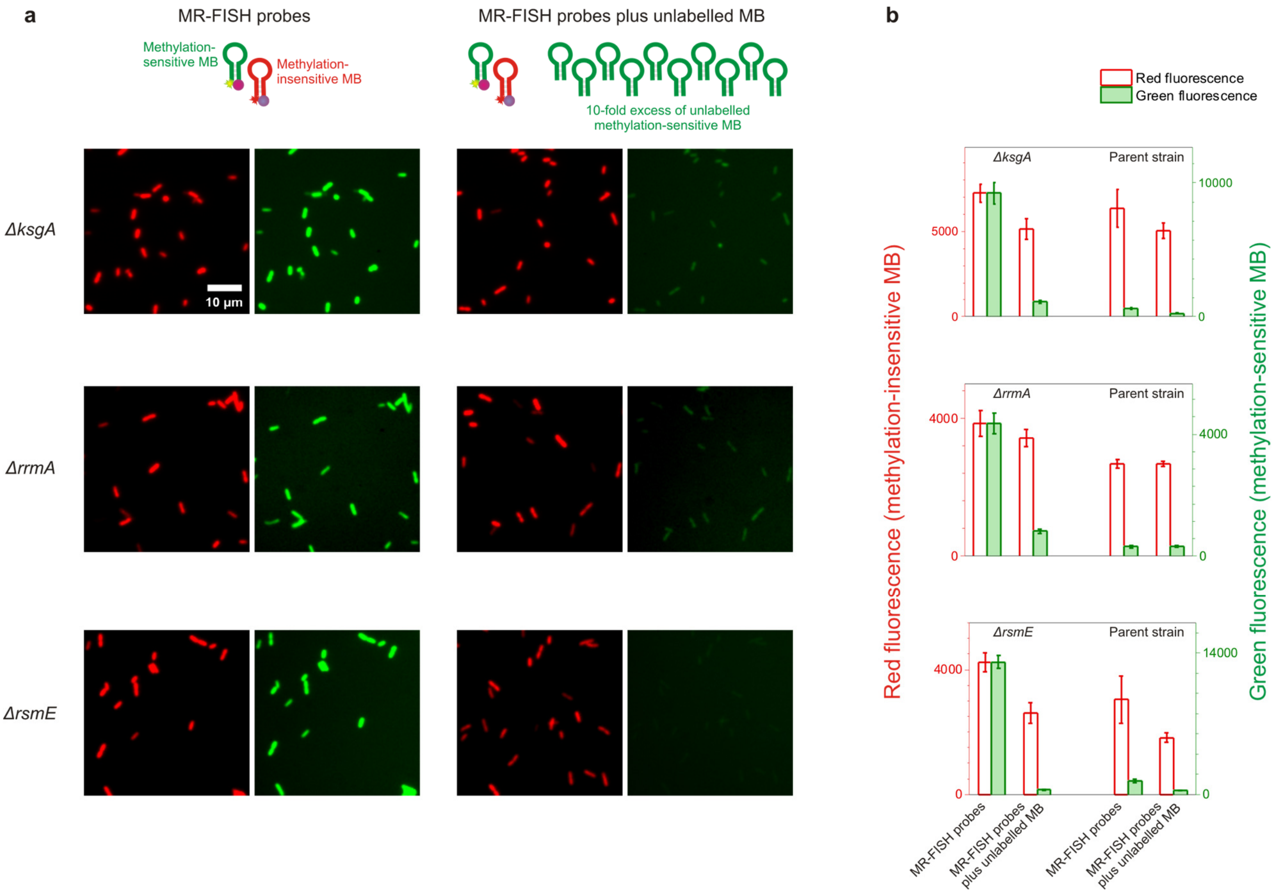

Supplementary Figure 10. Reduction of signal with competitor oligonucleotides shows that fluorescence results from hybridization. (a) False-colored fluorescence images (red: left panel of each pair; green: right panel of each pair) of methyltransferase deletion mutant bacteria stained by MR-FISH in the absence and presence of a 10-fold excess of unlabelled methylation-sensitive MBs. For each cell type, both red images are shown at one fixed brightness and contrast, and both green images at another fixed brightness and contrast. **(b)** Effect of excess unlabelled methylation-sensitive MBs on the mean red and green fluorescence of parent strain and methyltransferase deletion mutant bacteria. The mean and standard deviations of technical replicates ($n = 3$-6 samples; 529-2268 cells per sample) are plotted. The green fluorescence intensity is selectively depleted by unlabelled MBs, indicating that the signal results from hybridization.

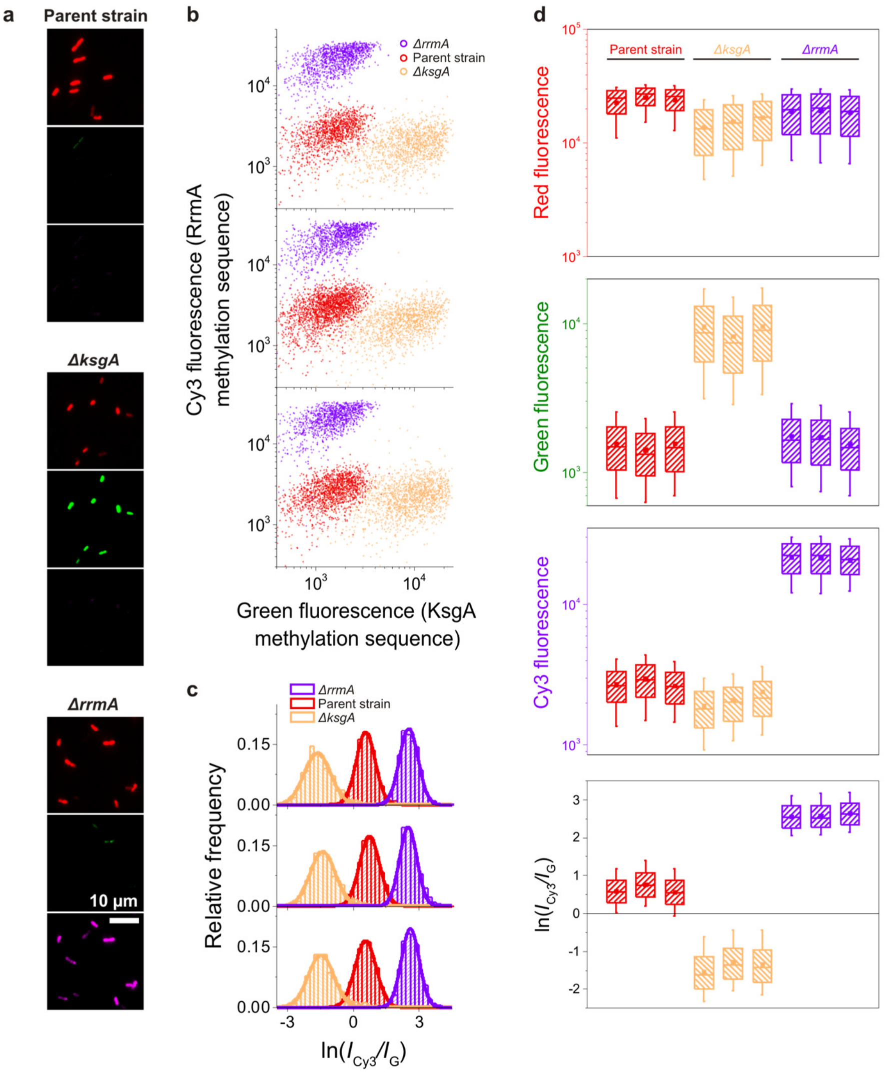

Supplementary Figure 11. Multiplexed detection of methylations in technical replicates. (a) Fluorescence images (top panel: Alexa Fluor 647, false colored red; second panel: Alexa Fluor 488, false colored green; third panel: Cy3, false colored magenta); showing that Δ*ksgA E. coli* are stained by Alexa Fluor-647 and Alexa Fluor 488-labeled MR-FISH probes, Δ*rrmA E. coli* are stained by Alexa Fluor-647 and Cy3-labeled MR-FISH probes, while parent-strain cells are brightly stained only by the methylation-insensitive Alexa Fluor-647-labeled probe. Scale bar: 10 µm. **(b)** Scatter plots of green and Cy3 fluorescence intensities of technical replicates of parent strain, Δ*ksgA* and Δ*rrmA* bacteria stained for 18 h [in each panel (from top to bottom), *n* = 1362 (parent strain), 1411 (Δ*ksgA*), and 1288 (Δ*rrmA*); 2095 (parent strain), 1447 (Δ*ksgA*), and 1207 (Δ*rrmA*); 1610 (parent strain), 1607 (Δ*ksgA*), and 1181 (Δ*rrmA*) cells per sample]. **(c)** Histograms of the log of the ratios of Cy3 fluorescence intensity (I_{Cy3}) to green fluorescence intensity (I_G) of technical replicates of parent strain, Δ*ksgA* and Δ*rrmA* shown in panel **(b)**. **(d)** Box plots of red fluorescence intensities, green fluorescence intensities, Cy3 fluorescence intensities and two-color ratios shown in panel **(b)** (boxes: 25th-75th percentiles; whiskers: 10th-90th percentiles; horizontal lines: medians; squares: means). As expected, the distributions of red fluorescence imparted by the methylation-insensitive Molecular Beacon overlap, while the fluorescence imparted by each of the methylation-sensitive Molecular Beacons is high only for the appropriate deletion strain.

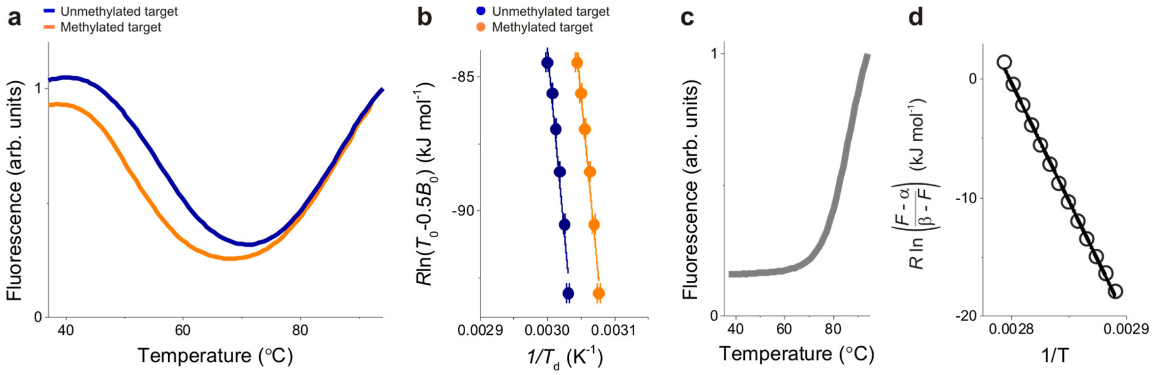

e

RNA Target	T_d	ΔT_d	$\Delta G_{1\text{-}2}$	$\Delta\Delta G_{1\text{-}2}$
UUGAACUCGCUGUGAAGAUGCAGUGUACCCG	58.5±0.02		45.0	
UUGAACUCGCUGUGm^{6}AAGAUGCAGUGUACCCG	53.7±0.1	4.8±0.1	40.5	4.5

f

Molecular Beacon sequence	T_b	$\Delta G_{2\text{-}3}$
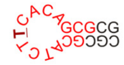	85.9±0.06	26.2

Supplementary Figure 12. Thermodynamic characterization of methylation-sensitive Molecular Beacon (MB) for m^{6}A. (a) Fluorescence melting curves of 2.5 µM methylation-sensitive beacon for m^{6}A (RlmJ MB) in the presence of 15 µM methylated and unmethylated RNA targets in 1× PBS with 1M NaCl. **(b)** Plots of $R\ln K$ vs. $1/T_d$ (the temperature at which the MB-RNA duplex is half-dissociated) at different RNA concentrations, used to calculate thermodynamic parameters of MB-RNA duplex dissociation. The mean and standard deviation (n = 5 technical replicates) of each data point is plotted. **(c)** Fluorescence melting curve of RlmJ MB (2.5 µM) in buffer (1× PBS with 1M NaCl). **(d)** Plot of $R\ln K$ vs. $1/T$ used to calculate thermodynamic parameters of MB dissociation. **(e)** Thermodynamic parameters determined for dissociation of RlmJ MB-RNA duplexes: T_d (at RNA concentrations of 15 µM, °C, mean ± s.e.m., n = 5 technical replicates) ΔT_d (°C, mean ± s.e.m.), $\Delta G_{1\text{-}2}$ (at 37 °C, kJ mol^{-1}) and $\Delta\Delta G_{1\text{-}2}$ (kJ mol^{-1}). Nucleotides complementary to MB sequences are shown in red. **(f)** Thermodynamic parameters determined for dissociation of RlmJ MB: T_b (°C, mean ± s.e.m., n = 5 technical replicates) and $\Delta G_{2\text{-}3}$ (at 37 °C, kJ mol^{-1}). Nucleotides complementary to RNA are shown in red, with the nucleotide complementary to m^{6}A is shown bold and underlined.

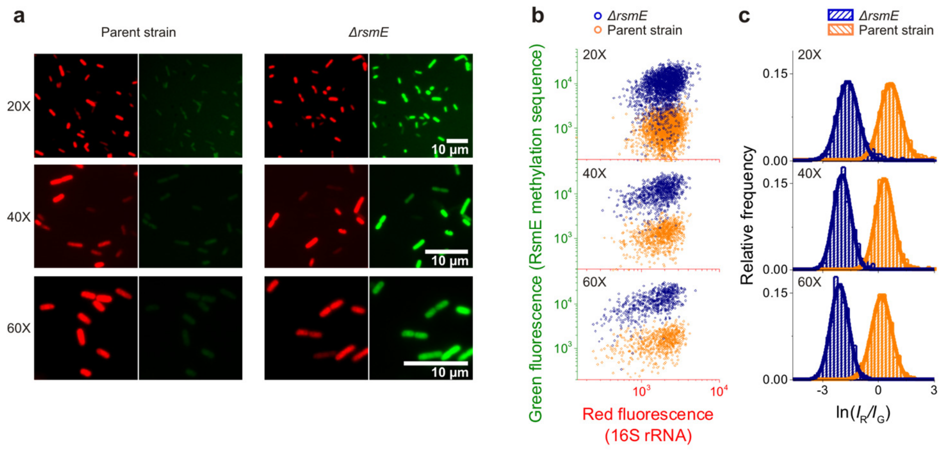

d

Microscope objective	Cell type	Cells detected	Number of Image frames	Cells per image frame
20X (NA = 0.5)	Parent strain	2542	60	42.4
20X (NA = 0.5)	*ΔrsmE*	2198	60	36.6
40X (NA = 0.95)	Parent strain	807	60	13.5
40X (NA = 0.95)	*ΔrsmE*	825	60	13.8
60X (NA = 1.4)	Parent strain	616	102	6.0
60X (NA = 1.4)	*ΔrsmE*	591	99	6.0

Supplementary Figure 13. Comparison of MR-FISH data collected using different microscope objectives. (a) False-colored fluorescence images (red: left panel of each pair; green: right panel of each pair) of parent strain and Δ*rsmE E. coli* collected with different microscope objectives [20× magnification, numerical aperture (NA) 0.5; 40× magnification, NA 0.95; or 60× magnification, NA 1.4]. **(b)** Scatter plots of red and green fluorescence intensities of parent strain and Δ*rsmE* bacteria collected with different microscope objectives. Numbers of cells per data set are given in panel **(d)**. **(c)** Histograms of the log of the ratios of red fluorescence intensity (I_R) to green fluorescence intensity (I_G) of parent strain and Δ*rsmE* bacteria in panel **(b)**. **(d)** Table showing the numbers of cells detected with each objective used to generate panels **(b)** and **(c)**. While the discrimination of parent strain and Δ*rsmE* bacteria by two-color ratio are similar with all objectives used, the throughput is greatest with the lowest-magnification 20× objective, as expected.

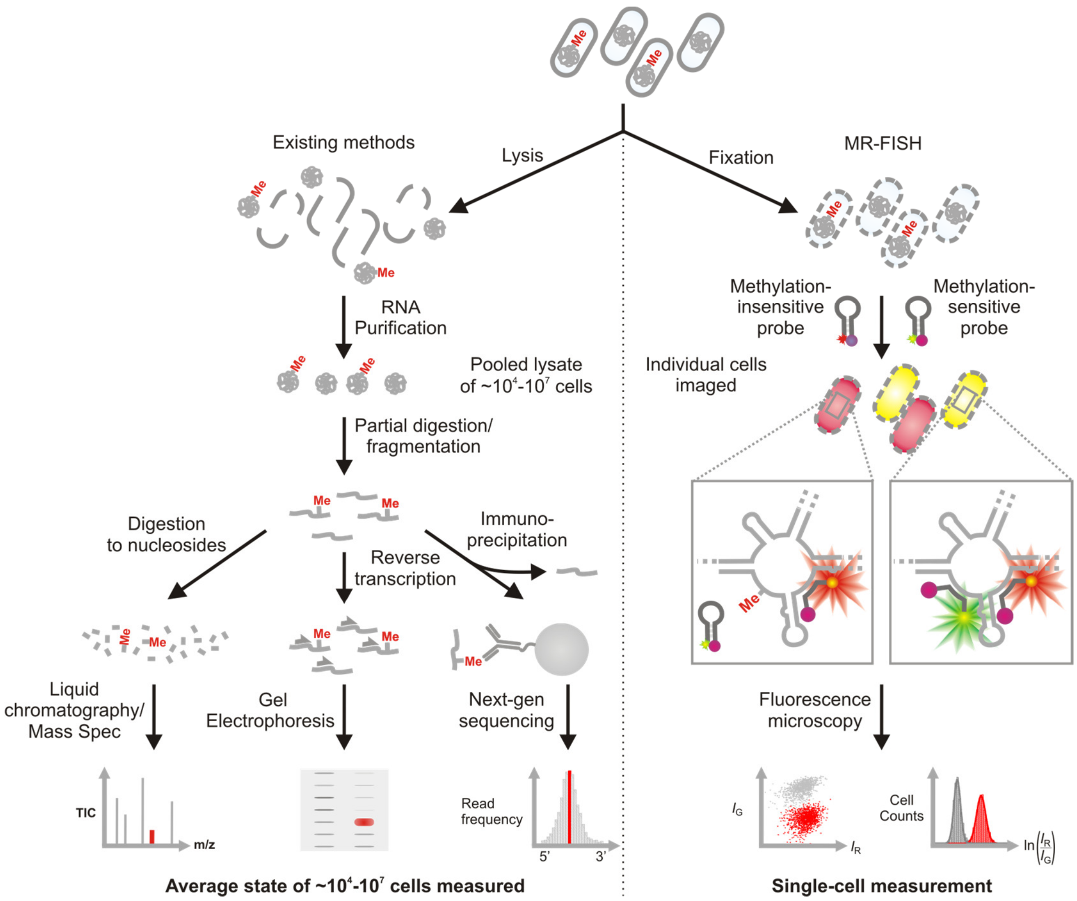

Supplementary Figure 14. Comparison of MR-FISH to existing methods for detecting base methylations.
